# Structural insights into HetR−PatS interaction involved in cyanobacterial pattern formation

**DOI:** 10.1038/srep16470

**Published:** 2015-11-18

**Authors:** Hai-Xi Hu, Yong-Liang Jiang, Meng-Xi Zhao, Kun Cai, Sanling Liu, Bin Wen, Pei Lv, Yonghui Zhang, Junhui Peng, Hui Zhong, Hong-Mei Yu, Yan-Min Ren, Zhiyong Zhang, Changlin Tian, Qingfa Wu, Mikael Oliveberg, Cheng-Cai Zhang, Yuxing Chen, Cong-Zhao Zhou

**Affiliations:** 1 Hefei National Laboratory for Physical Sciences at the Microscale and School of Life Sciences, University of Science and Technology of China, Hefei Anhui 230027, China; 2 Department of Biochemistry and Biophysics, Arrhenius Laboratories of Natural Sciences, Stockholm University, S-106 91 Stockholm, Sweden; 3 Aix Marseille Université and Laboratoire de Chimie Bactérienne, Unité Mixte de Recherche 7283, Centre National de la Recherche Scientifique, 31 Chemin Joseph Aiguier, 13402 Marseille Cedex 20, France

## Abstract

The one-dimensional pattern of heterocyst in the model cyanobacterium *Anabaena* sp. PCC 7120 is coordinated by the transcription factor HetR and PatS peptide. Here we report the complex structures of HetR binding to DNA, and its hood domain (HetR_Hood_) binding to a PatS-derived hexapeptide (PatS6) at 2.80 and 2.10 Å, respectively. The intertwined HetR dimer possesses a couple of novel HTH motifs, each of which consists of two canonical α-helices in the DNA-binding domain and an auxiliary α-helix from the flap domain of the neighboring subunit. Two PatS6 peptides bind to the lateral clefts of HetR_Hood_, and trigger significant conformational changes of the flap domain, resulting in dissociation of the auxiliary α-helix and eventually release of HetR from the DNA major grove. These findings provide the structural insights into a prokaryotic example of Turing model.

Pattern formation is a fundamental process in developmental biology not only in plants and animals but also in those prokaryotes capable of multicellular behaviors. Although developmental pattern requires complex regulatory networks, basic regulatory modules fulfilling the requirement of the Turing model can be used to explain the occurrence of spatial morphological patterns^1^,^2^. One simple example of pattern formation coordinated by the activator-inhibitor pair was found in the filamentous cyanobacterium *Anabaena* sp. PCC 7120 (hereafter *Anabaena*)^3^. In the absence of combined nitrogen, about 5–10% of the cells along the *Anabaena* filament can differentiate into highly specialized cells, called heterocysts, specialized for dinitrogen fixation^4^. Heterocysts are regularly intercalated among vegetative cells, forming a one-dimensional pattern^5^. Development of heterocysts, as well as the establishment and the maintenance of this regular pattern, is finely regulated by a signaling cascade^6^. This cascade is mainly coordinated by two transcription factors, NtcA and HetR (Fig. 1). Deprivation of combined nitrogen leads to the accumulation of 2-oxogluatarate, which in consequence activates NtcA to trigger the differentiation of heterocysts^7^,^8^. Notably, both NtcA and HetR are auto-regulated and mutually dependent on each other for expression^9^. While NtcA is a global regulator that initiates the heterocyst differentiation, HetR is a master regulator that controls the heterocyst development and pattern formation^10^. HetR dimer specifically binds to the promoter regions of *hetR* and *patS*^11^, suggesting an autoregulation and Turing network (Fig. 1). After a 17-bp partially palindromic HetR-binding sequence was clearly defined within the promoter of heterocyst-related gene *hetP*^12^, hundreds of putative HetR regulatory sites in the *Anabaena* genome have been proposed by transcriptomic approaches and bioinformatics analyses^13^,^14^; however, only 38 direct HetR-binding sites were identified by the chromatin pull-down combined with deep sequencing^15^.

Heterocyst pattern was first speculated to be controlled by a pentapeptide RGSGR (designated as PatS5), a motif which was identified first as the C-terminal five residues of PatS^16^, and later within a putative ketoacyl reductase HetN^17^. Binding of PatS5 to HetR may abolish the DNA-binding capacity of HetR^11^, a proposed mechanism to prevent the occurrence of multiple contiguous heterocysts (Fig. 1). The *in vitro* binding assays suggested that two PatS5 molecules bind to a HetR homodimer at a dissociation constant *K*_*d*_ of 227 nM^18^, most likely at the proximity of the C-terminal region^18^. Notably, HetR has a higher affinity towards a hexapeptide ERGSGR (termed PatS6, at a *K*_*d*_ of 7 nM), compared to that of PatS5^19^. However, the *in vivo* experiments suggested that the last eight residues could recreate the full activity of the native PatS^20^. Thus the bona fide active form of PatS under physiological conditions remains unknown.

The action of HetR and PatS in heterocyst pattern formation is reminiscent of those regulatory modules giving rise to Turing patterns in embryonic development^2^,^21^. The autoregulation of HetR provides a short-range positive feedback and activates the expression of *patS*, in turn PatS peptide acts as a long-range negative feedback by lateral diffusion among neighboring vegetative cells and inhibits the activity of HetR (Fig. 1). However, since the mechanism by which PatS acts on HetR has not yet been understood, we still lack major parameters to test experimentally the usefulness of the Turing model for heterocyst pattern formation. Indeed, no structural studies have been successful on HetR of *Anabaena* despite attempts of several laboratories^22^,^23^, although Kim *et al.* reported the structures of the thermophilic cyanobacterium *Fischerella* HetR apo-form and complexes with palindromic DNA of various lengths derived from *Anabaena hetP* promoter^23^,^24^. The *Fischerella* HetR forms an intertwined homodimer, each subunit of which consists of three distinct domains: an N-terminal DNA-binding domain (DBD) followed by a flap domain and a C-terminal hood domain. Nevertheless, how DNA is released from HetR in the presence of the mature form PatS remains unknown. Here we report the crystal structure of *Anabaena* HetR in complex with the 21-bp DNA of *hetP* promoter, and the complex structure of the hood domain with PatS6. Comparative structural analyses, together with computational simulations and biochemical assays enable us to suggest a putative mechanism of DNA binding to and release from HetR.

## Results

### Overall structure of *Anabaena* HetR binding to *hetP* promoter

**Figure 2:**
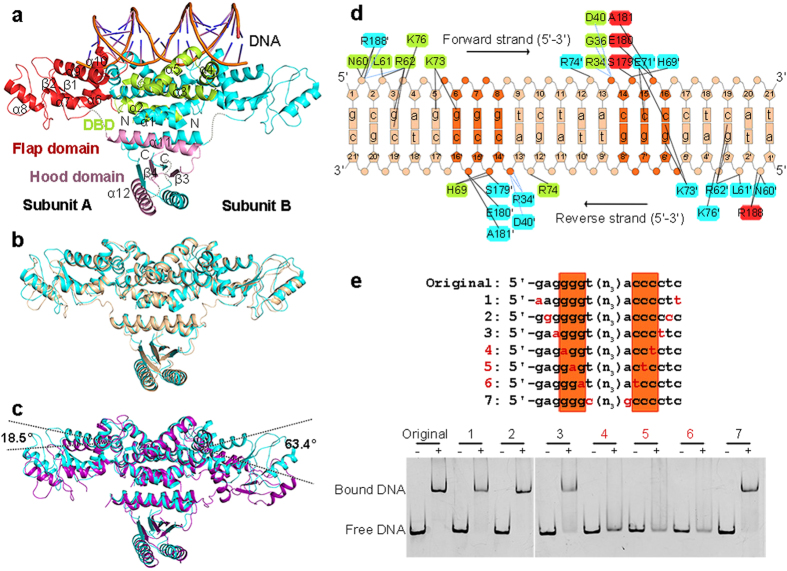
Structure of HetR−DNA complex. (**a**) Overall structure of *Anabaena* HetR dimer in complex with the 21-bp DNA from the *hetP* promoter. The secondary structural elements of DBD (limon), flap domain (red) and hood domain (pink) in subunit A are labeled. The missing residues are depicted as the dashed lines. Comparison of DNA-bound *Anabaena* HetR (cyan) with (**b**) the DNA-bound (wheat) and (**c**) apo-form (purple) *Fischerella* HetR. The rotation angles of the flap domains are labeled. (**d**) Schematic representation of interaction networks between *Anabaena* HetR and DNA. Interacting residues are marked with the same color as their corresponding domains, respectively. Direct hydrogen bonds are indicated as black lines and water-mediated hydrogen bonds as blue dashes. (**e**) EMSA assays of HetR with variations of DNA. DNA samples with and without HetR are shown as “+” and “−”, respectively. The corresponding DNA variations numbered as No.1 to 7. The 5′-g^6^gg and 5′-c^14^cc motifs are highlighted in dark orange.

In the refined model of HetR−DNA complex, the 21-bp double-stranded DNA is clearly traced, whereas several regions in the structure of HetR (residues Ala216–Asp220 and Gly284–Gly285 in subunit A, Ala216–Gln221 and Gly284–Ile286 in subunit B) are missing. Each asymmetric unit contains an intertwined, compact HetR dimer and a 21-bp duplex DNA 5′-gcgaggggtctaacccctcat (Fig. 2a). The two subunits run across each other to form a highly entangled dimer, with a buried interface area of about 7100 Å^2^ on each side. The N-terminal and C-terminal segments contribute to the majority of interface, whereas the central segment protrudes outwards like two flaps. The hydrophobic interactions between the crossing-over N-terminal α-helices (α1–5) of the two subunits might be the driving force for the folding of HetR dimer.

Similar to the structures of *Fischerella* HetR^23^,^24^, each *Anabaena* HetR subunit contains three distinct domains: the N-terminal DBD (residues 1–98), the middle flap domain (residues 99–216), and a slightly smaller C-terminal hood domain (residues 217–299) (Fig. 2a). The DBD has a HTH motif (α4 and α5) that inserts into the major groove of DNA. Notably, the partially palindromic DNA sequence in our structure is exactly the same as the HetR recognition sequence in the *hetP* promoter^12^, whereas the DNA sequence in the previous *Fischerella* HetR structures is derived from the *hetP* promoter region but modified to be perfectly palindromic^24^. Due to a sequence identity of 90% between *Anabaena* and *Fischerella* HetR proteins, the two DNA-complexed structures (PDB 4YRV and 4IZZ) are quite similar to each other with a root-mean-square deviation (RMSD) of 1.0 Å over 462 Cα atoms. In both structures, HetR adopts an active conformation, with the two 33.0 Å-apart HTH motifs perfectly accommodated in a successive DNA major groove. Moreover, the two flap domains in both complex structures adopt the same conformation and orientation (Fig. 2b), which are stabilized by the duplex DNA via interactions with the two α10 helices.

Comparison of *Anabaena* HetR−DNA complex (PDB 4YRV) with the apo-form *Fischerella* HetR (PDB 3QOD)^23^ reveals significant conformational changes upon DNA binding. Both the DBDs and the hood domains are well superimposed, whereas the two flap domains vary dramatically (Fig. 2c). Upon the DBDs binding to DNA, the flap domains rotate as a rigid body towards DNA 18.5° for subunit A or 63.4° for subunit B (Fig. 2c), resulting in a decreased distance between the two HTH motifs from 36.3 to 33.0 Å.

Distinct from the previously reported HTH motif^25^, the DNA-binding motif of HetR is composed of three helices: the two canonical helices α4 and α5, in addition to an auxiliary helix α10 from the symmetric subunit. Moreover, both the flap and the hood domains adopt novel folds; thus HetR represents a novel transcription factor.

### The DNA-binding pattern of *Anabaena* HetR

HetR dimer and DNA duplex form a complex at a molar ratio of 1:1. The 21-bp duplex DNA adopts a right-handed B-form conformation, with a total bent angle of 33.9°, as calculated by the Curves+ web server^26^. The palindromic sequences 5′-g^6^gg and 5′-c^14^cc have a larger major groove of 13.3 and 8.2 Å in width and depth, respectively, compared to the ideal B-DNA (10.5 and 5.4 Å, respectively). The two HTH motifs, which are positively charged due to clustering of the basic residues Arg62, His69, Lys73, Arg74 and Lys76, are complementary to the duplex DNA palindrome. Notably, the two interfaces between DNA and HetR dimer are not perfectly symmetric (Fig. 2d), owing to the imperfect palindromic sequence of *hetP* promoter. Besides the shared specific interactions of g3-Arg62 and g6-Lys73 at both interfaces, the base g5 forms a hydrogen bond with Lys73 at one interface, whereas bases c15 and c16 form two hydrogen bonds with Glu71 at the other interface. In addition, a cluster of nonspecific interactions between HetR and the sugar-phosphate backbone of DNA, together with water-mediated hydrogen bonds further stabilize the HetR−DNA complex (Fig. 2d).

The 5′-g^6^gg and 5′-c^14^cc motifs flanking the center of DNA contribute to the majority of interactions with HetR (Fig. 2d), indicating a DNA consensus of 5′-gggn_5_ccc. To assess the contribution of each base in the consensus sequence, we used electrophoretic mobility shift assays (EMSA) to compare the HetR-binding affinities of corresponding variations with the original sequence (Fig. 2e). Among the seven variations, DNA sequences No. 4, 5 and 6, each of which possesses a pair of transitions at 5′-g^6^gg and 5′-c^14^cc, totally lost the binding capacity towards HetR, whereas the other four variations remain a comparable HetR-binding affinity with the original sequence. In addition, alterations of any other bases beyond the consensus have no detectable change of DNA-binding capacity (Supplementary Fig. 1). Together, we conclude that the central motif of 5′-gggn_5_ccc is indispensable for binding to HetR.

### The PatS6-binding site

**Figure 3:**
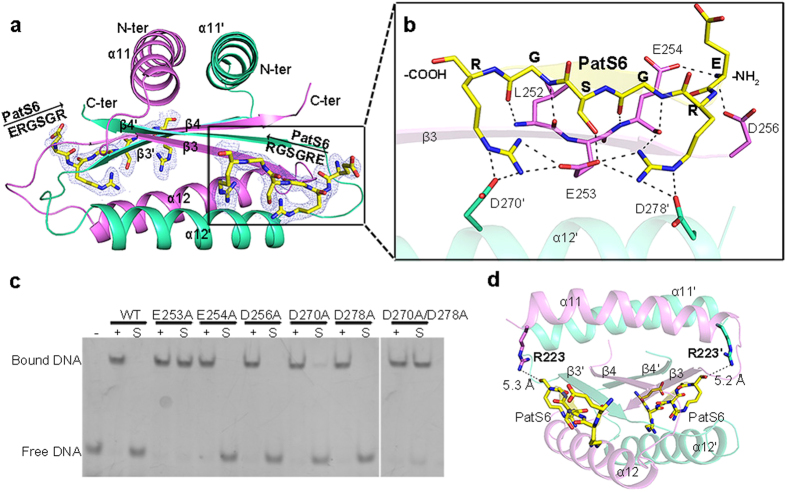
PatS6-binding mode. (**a**) Overall structure of the PatS6−HetR_Hood_ dimer. Two PatS6 peptides (yellow) are shown as sticks, with the *F*_*o*_ − *F*_*c*_ electron-density omit map contoured at 3.0 sigma. (**b**) A close-up view of the PatS6-binding site. Hydrogen bonds and the polar interactions are indicated as dashed lines. (**c**) EMSA assays of HetR and mutants with original DNA sequence, in the presence or absence of PatS6. “−” represents DNA probe without protein. “+” indicates DNA incubated with HetR or mutants, whereas “S” indicates addition of PatS6 to the HetR−DNA complex. (**d**) The distance between Arg223 and the C-terminus of PatS6 in each subunit is labeled.

Based on the structures of *Fischerella* HetR, three potential PatS5 binding sites have been proposed^23^. In addition, electron paramagnetic resonance spectroscopy further suggested that PatS5 may bind to a region in the hood domain^18^. However, it lacks a direct structural evidence for the precise binding site. To assign the precise binding site of PatS peptide, we performed the co-crystallization experiments using the peptides PatS5 and PatS6 of higher *in vitro* binding affinity^19^. In an initial attempt to get the complex structure of PatS−HetR, we found that the addition of either PatS5 or PatS6 led to the precipitation of HetR in solution. As seen from the HetR−DNA complex structure, the hood domain is somewhat independent of the other two domains (Fig. 2a). Thus we overexpressed and applied the individual hood domain (Asp219-Asp299, termed HetR_Hood_) of HetR for co-crystallization with PatS5 or PatS6. While the crystallization trials with PatS5 yielded no diffraction-quality crystals, we successfully solved the crystal structure of HetR_Hood_ complexed with PatS6 (termed PatS6−HetR_Hood_) at 2.10 Å resolution (Table 1). Each asymmetric unit contains four molecules, which form two HetR_Hood_ dimers that are nearly identical to each other with an RMSD of 0.8 Å over 165 aligned Cα atoms. The two subunits of HetR_Hood_ dimer also adopt an α-β-α-β topology to form an entwined dimer (Fig. 3a), similar to the counterparts in HetR−DNA complex structure. PatS6 binds to the lateral cleft of hood domain in each subunit via four main-chain hydrogen bonds with β3, forming an extended antiparallel β-sheet of six β-strands (Fig. 3b). Besides, the two arginine residues of PatS6 make four hydrogen bonds with the carboxyl group of Glu253 in β3, in addition to four hydrogen bonds with Asp270′ and Asp278′ of α12′, whereas the serine residue of PatS6 forms a hydrogen bond with Glu253. Notably, the N-terminal amino group of PatS6 is captured by the side chains of Glu254 and Asp256 from both sides, resulting in a higher affinity of PatS6 compared to PatS5^19^. However, substitution of this N-terminal residue did not affect the HetR binding affinity^19^, for the interactions with HetR come from only the main-chain amino group, but independent of the side chain, of the N-terminal glutamate of PatS6. As seen from Fig. 3a, the N-terminus of PatS6 is somewhat restricted by the loop between α12 and β4; thus, extension of the N-terminal to 7 or more residues should introduce a significant steric hindrance and lead to a lower affinity^19^. The structural information, together with previous biochemical characterization^19^, indicates that the hexapeptide with a conserved sequence of XRGSGR (X stands for any residue) is most likely a mature form of PatS. Further sequence analyses suggested that these PatS6-binding residues are strictly conserved among different species of heterocyst-forming cyanobacteria (Supplementary Fig. 2).

To validate the PatS6-binding mode, we determined the binding affinities of PatS6 towards the wild-type HetR or mutants. PatS6 showed a *K*_*d*_ of 12 nM towards the wild-type HetR, similar to the previous report of Feldmann *et al.*^19^. Mutation of E253A totally abolished the PatS6-binding affinity, suggesting its indispensable role. Single mutation of E254A, D256A, D270A or D278A led to a dramatically lower binding affinity (Supplementary Fig. 3), with a *K*_*d*_ of 91, 427, 2252 or 1626 nM, respectively, whereas the double mutant D270A/D278A showed no detectable binding affinity. In fact, *in vivo* experiments of strains with a *hetR* allele coding for conservative substitutions at residues Glu253–Asp256 showed an altered percentage of heterocysts; moreover the E253D mutant strain had a drastically reduced sensitivity to PatS5^18^.

To check if these PatS6-binding residues also contribute to the release of DNA from HetR upon binding to PatS6, we applied the above mutants to EMSA assays (Fig. 3c). First, all mutants exhibited a DNA-binding capability as strong as the wild-type HetR, indicating these residues are not directly involved in DNA binding. The addition of excess PatS6 could trigger the dissociation of DNA from the wild-type HetR and mutants E254A, D256A, D270A and D278A, suggesting the signal of PatS6 binding could be transferred from the hood domain to the DBD in these mutants. However, the DNA-binding capability of mutant E253A or double mutant D270A/D278A is independent of the addition of PatS6, indicating that Glu253 in β3 and residues Asp270&Asp278 are indispensable for transferring the signal of PatS6 binding.

Notably, an R223W mutant of HetR was shown to be insensitive *in vivo* to the overexpressed PatS or HetN^27^, indicating that Arg223 might also participate in PatS6 inhibition. Indeed, the R223W mutant has a *K*_*d*_ value of 2353 nM towards PatS6 (Supplementary Fig. 3), indicating a much lower binding affinity compared to the wild-type HetR. In the PatS6−HetR_Hood_ complex structure, the side chain of Arg223 is very close to the electronically complementary C-terminal carboxyl group of PatS6 (Fig. 3d). Therefore, replacement of the negatively-charged Arg223 with a highly hydrophobic tryptophan should abolish the binding towards PatS6. In addition, ITC assays showed that PatS6 has a much lower binding affinity towards HetR_Hood_ (*K*_*d*_ of 24 μM), compared to that of the full-length HetR with a *K*_*d*_ value of 12 nM (Supplementary Fig. 3). It indicated that the other domains of HetR also influence the binding affinity towards PatS6.

### The flap domains undergo significant conformational changes upon binding to PatS6

Comparison of the overall structures of hood domain in PatS6−HetR_Hood_ and HetR−DNA complex reveals slight conformational changes (with an RMSD of 0.7 Å over 145 Cα atoms). To understand how these slight signals are amplified and transferred to the DBD, we performed 20-ns molecular dynamics simulations with the HetR−DNA and modeled PatS6−HetR−DNA complex respectively, using the enhanced sampling method^28^.

**Figure 4:**
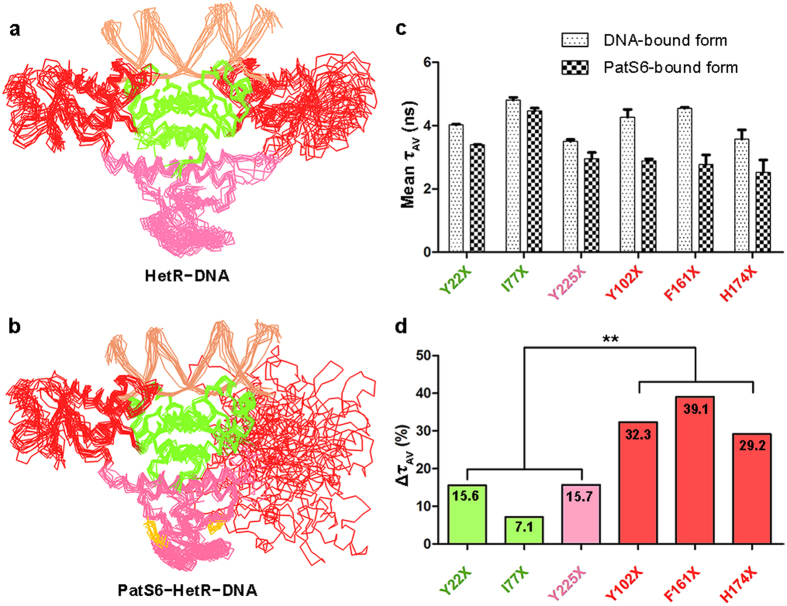
Putative conformational changes of HetR upon PatS6 binding. Computational simulation of (**a**) HetR−DNA and (**b**) PatS6−HetR−DNA. The structure ensembles, including DNA (light orange) and PatS6 (yellow), are presented as ribbon. (**c**) Fluorescence lifetime spectroscopy assays of six HetR mutants. The amplitude-averaged lifetimes (τ_AV_) of six HetR mutants with standard error are shown as histograms. (**d**) The relative changes of τ_AV_ in percentage. The data are analyzed using an independent samples t-test (**stands for p < 0.01).

During the 20-ns simulation, the HetR−DNA complex remains in its original state, as reflected by the average RMSD of 3.6 Å over 598 Cα atoms (Supplementary Fig. 4). Ten representative snapshots with equal time intervals were superimposed against the original HetR−DNA structure with the DBDs aligned and revealed slight conformational changes, which indicated that the HetR−DNA complex was relatively stable during the 20-ns simulation (Fig. 4a). In contrast, the PatS6−HetR−DNA complex showed drastic conformational changes during the 20-ns simulation with an average RMSD of 12.1 Å over 598 Cα atoms (Supplementary Fig. 4). Superposition of the 10 snapshots of PatS6−HetR−DNA complex suggests that the flap domain in subunit B waggles dramatically (Fig. 4b). Despite PatS6 binding triggers only slight local conformational changes at the hood domain, the signal might be amplified through the overall structure of HetR, resulting in a higher flexibility of the flap domains and eventually the release of DNA from the DBDs.

To prove this hypothesis, we performed fluorescence lifetime spectroscopy assays using six HetR mutants, Y22X, I77X, Y225X, Y102X, F161X or H174X, where X stands for the rac-(7-hydroxycoumarin-4-yl) ethylglycine (hereafter 7-HC), a fluorescent probe which is sensitive to small polarity changes since its fluorescence lifetime is highly influenced by the polarity of their microenvironment^29^. To compare the flexibility of the three domains of HetR upon PatS6 binding, residues Tyr22 and Ile77 from the DBD, Tyr225 from the hood domain, and Tyr102, Phe161 and His174 from the flap domain were applied to the mutageneses. EMSA assays confirmed that all these mutants maintained the DNA-binding capability as well as the sensitivity towards PatS6 (Supplementary Fig. 5). The amplitude-averaged lifetimes (τ_AV_) of 7-HC in DNA and PatS6-bound form for each HetR mutant are shown in Fig. 4c. The mean τ_AV_ in the DNA-bound form of HetR mutants Y22X, I77X, and Y225X are 4.0, 4.8, and 3.5 ns, respectively. Upon the addition of PatS6 in the HetR−DNA solution, the values were slight decreased to 3.4, 4.5, and 3.0 ns, respectively, resulting in a relative decrease of τ_AV_ of 15.6%, 7.1% and 15.7%, respectively. In contrast, the three mutations Y102X, F161X and H174X in the flap domain gave a decrease of τ_AV_ at 32.3%, 39.1% and 29.2%, respectively, significantly higher than the former three mutations, which are in the DBD or hood domain (Fig. 4d). It is in agreement with the hypothesis that the flap domains undergo significant conformational changes upon binding to PatS6.

## Discussion

Oligopeptides have long been recognized as a kind of transcription regulators, either activating or repressing transcription. The regulatory peptides could repress transcription in a variety of ways, for example, inhibiting kinase activity of the sensor in a two-component system^30^, blocking the secondary channel of RNA polymerase^31^, or preventing the dimerization of transcription factors via competitive binding^32^. In contrast, peptide-induced conformational changes of transcription factor have only been reported in the cases of transcriptional activation^33^,^34^. Thus interactions between PatS and HetR represent an example of allosteric inactivation of a transcription factor upon peptide binding.

**Figure 5:**
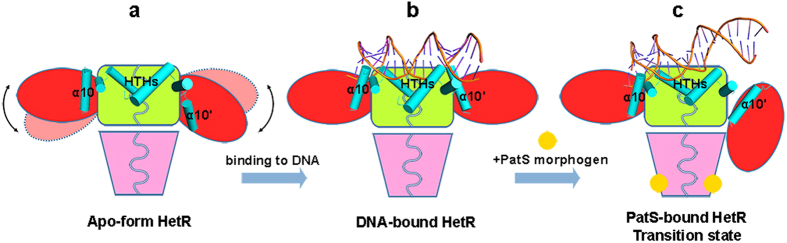
A proposed inhibition mechanism of HetR upon binding to PatS. The duplex DNA is shown as a cartoon, and PatS as a yellow sphere. The HTH motifs, including the two auxiliary α10 helices, are shown as cylinders.

Based on the structural analyses and computational simulations, we propose a mechanism for DNA binding to and release from HetR. In the apo-form state (Fig. 5a), HetR forms a stable dimer with the DBD and hood domains extensively entangled, whereas the two flap domains show a relatively plastic conformation of slight fluctuations. Notably, previous structures of *Fischerella* HetR suggested that HetR adopts a dimeric or tetrameric conformation, upon binding to DNA fragments of different lengths^24^. However, HetR forms a dimer in both the previous and our present structures complexed with a 21-bp DNA. Upon binding to a target promoter region, the two HTH DNA-binding motifs, together with two auxiliary α10 helices from the flap domains, capture the duplex DNA, which in turn freeze the two flap domains at a stable conformation (Fig. 5b), that ultimately activates the transcription of target genes involved in heterocyst differentiation, including *patS*^11^. Afterwards, the expression of *patS*, followed by that of *hetN,* synthesizes the PatS precursors, which are subject to the process of maturation to form the common PatS morphogen. As a small molecule, PatS diffuses from differentiating heterocysts to the neighboring vegetative cells along the filament. In vegetative cells, binding of PatS to the hood domain of HetR initially triggers the conformational changes of the flap domain, as seen from the computational simulations, resulting in the dissociation of one α10 helix from the DNA major groove (Fig. 5c), eventually the instability of the HetR−DNA complex, and finally the complete release of DNA from HetR and termination of transcription. This finely regulated cascade that controls the heterocyst pattern formation represents another example of Turing model.

## Methods

### Protein expression, purification and crystallization

The *hetR* gene from *Anabaena* sp. PCC 7120 was cloned into a modified pET28 vector with a six-histidine tag at the N-terminus. The DNA sequence encoding HetR_Hood_ (Asp219-Asp299) was also cloned into this pET28a-derived vector. The two recombinant plasmids were validated by DNA sequencing (Sangon Biotech, Shanghai). HetR and HetR_Hood_ proteins were overexpressed in *Escherichia coli* strain Rosetta (DE3) (Novagen, Madison). Cells were grown in 2 × YT medium (5 g NaCl, 16 g Bacto-Tryptone, and 10 g yeast extract per liter) at 37 °C containing kanamycin and chloramphenicol at 30 and 34 μg/ml, respectively. At an OD_600 nm_ of 0.8, protein expression was induced with 0.2 mM isopropyl β-D-1-thiogalactopyranoside at 16 °C for 20 hr. Cells were harvested by centrifugation (6,000 × g, 4 °C, 10 min) and resuspended in 40 ml lysis buffer (1 M NaCl, 10 mM Tris-Cl, pH 7.8). After 5 min of sonication and centrifugation at 12,000 × g for 30 min, the supernatant containing the soluble target protein was pooled and loaded onto a Ni-NTA column (Qiagen, Mississauga, ON) equilibrated with the binding buffer (1 M NaCl, 10 mM Tris-Cl, pH 7.8). The target protein was eluted with 300 mM imidazole, and further applied to a Superdex 75 column (GE Healthcare, UK) equilibrated with the binding buffer. The purity of protein was assessed by gel electrophoresis and the protein sample was stored at −80 °C.

The selenium-methionine-labeled HetR (SeMet HetR) was expressed in *E. coli* strain B834 (DE3) (Novagen, Madison). Transformed cells were first cultured in 2 × YT medium at 37 °C overnight, then harvested and washed twice with the M9 medium. Then the cells were cultured in SeMet medium (M9 medium with 50 mg/l SeMet and other essential amino acids at 50 mg/l) to an OD_600_ _nm_ of approximately 1.0. The following steps in protein expression and purification were the same as those for the native protein. Site-directed mutagenesis was performed using the QuickChange site-directed mutagenesis kit (Stratagene, La Jolla) with the plasmid encoding the wild-type HetR as the template. The mutant protein was expressed, purified and stored in the same manner as the wild-type protein.

The protein for crystallization was concentrated to 12 mg/ml by ultrafiltration (Millipore Amicon). The single-stranded DNA was synthesized by Sangon Biotech. Complementary DNA strands were heated at 95 °C for 5 min and then annealed slowly to room temperature. Prior to crystallization, DNA duplex and the recombinant HetR were mixed in a 1.1:1.0 molar ratio, whereas HetR_Hood_ was incubated with 8 mM PatS6 (ERGSGR) synthesized by GL Biochem (Shanghai). All crystals were grown using the hanging drop vapor diffusion method at 13 °C. The nanopipetting was performed using the Mosquito nanoliter liquid handling system (TTP LabTech). The native HetR–DNA crystals were obtained against the reservoir solution of 0.3 M calcium acetate and 0.1 M Bicine, pH 9.0, while the SeMet HetR–DNA crystals were grown against 0.2 M magnesium acetate for two days. The native PatS6–HetR_Hood_ crystals were obtained from 25% PEG 4000, 0.1 M sodium citrate, pH 5.6 and 0.2 M ammonium acetate. All the crystals were transferred to the cryoprotectant (reservoir solution supplemented with 30% glycerol) and flash-cooled in liquid nitrogen before data collection.

### Data collection and processing

X-ray diffraction data were collected at 100 K in a liquid nitrogen stream, using beamline BL17U with a Q315r CCD (ADSC, MARresearch, Germany) at the Shanghai Synchrotron Radiation Facility (SSRF). All diffraction data were integrated and scaled with the program HKL2000^35^.

### Structure determination and refinement

The selenium sites from the data of SeMet HetR–DNA complex were determined using the program SHELXD^36^. The initial phases were calculated with the program OASIS^37^, then improved with the programs RESOLVE and Buccaneer^38^,^39^,^40^. Autobuild in PHENIX^41^ was used to perform automatic model building. The initial model was refined by REFMAC5^42^. The corresponding modules of SeMet HetR–DNA complex were used as the search model against the native data of HetR–DNA and PatS6–HetR_Hood_ by molecular replacement using Molrep program as part of CCP4i^43^ program suite. The models were refined by REFMAC5 and rebuilt interactively with the programs PHENIX (or REFMAC5) and COOT^44^ until the free R-factor converged. Crystallographic parameters were listed in Table 1. All final models were evaluated with the programs MOLPROBITY^45^ and PROCHECK^46^. The final refined models of HetR–DNA/PatS6–HetR_Hood_ had reliable geometry, with 92.4/99.4% of the amino acid residues in the favored regions and 0.7/0.0% residues in the disallowed regions of the Ramachandran plot, respectively.

### Isothermal titration calorimetry (ITC) assays

Purified HetR and mutants were dialyzed against a buffer containing 1 M NaCl, 5% (v/v) glycerol, and 10 mM Tris-Cl, pH 7.8 for 12 hr. The data were collected on an iTC200 (MicroCal) at 25 °C by injecting an initial 0.4 μl aliquot and the following 19 consecutive 2 μl aliquots. The sample cell was loaded with 200 μl protein while the injection syringe was loaded with 40 μl PatS6. The wild-type HetR, mutants E254A and D256A were diluted to a final concentration of 10 μM, whereas the concentration of mutants R223W, E253A, D270A, D278A, D270A/D278A and HetR_Hood_ was 50 μM. The concentration of PatS6 was 15 times (150 or 750 μM) to that of the full-length protein or 40 times (2 mM) to that of HetR_Hood_.

### EMSA assays

The oligonucleotides labeled with 6-carboxyfluorescein (also known as 6-FAM) at the 5′-end were synthesized by Sangon Biotech (Shanghai). The 29-bp and 120-bp double-stranded DNA probes were generated by annealing and PCR reactions, respectively. The DNA probes were incubated with the proteins at 4 °C for 30 min. The competitor poly(dI-dC) was added for eliminating the nonspecific DNA-binding. The final concentrations of proteins, DNA probes and PatS6 peptides were 2 μM, 50 nM and 100 μM, respectively. Then the mixture was separated by 8% native-PAGE.

### Molecular dynamics simulation with enhanced sampling method

To study the conformational changes of HetR, a previously developed enhanced sampling method^28^ was performed in molecular dynamics simulation, which can efficiently accelerate large-scale conformational changes of a multi-domain protein in a relatively short-time simulation. The simulation was set up using the modified GROMACS-4.5.5 package^47^ and the CHARMM27 force field^48^. The HetR–DNA complex (or the modeled PatS6–HetR–DNA complex) was first placed in a rhombic dodecahedron box, with the minimum distance between the solute and the box boundary of 1.2 nm. TIP3P water molecules^49^ were then filled the box. The energy of the system (DNA, protein and waters) was minimized by the steepest descent method, until the maximum force was smaller than 1500 kJ mol^−1^ nm^−1^. Sodium ions were added by replacing the same number of waters with the most favorable electrostatic potential, in order to compensate the net positive charges on the complex. The final system (DNA, protein, waters, and ions) was minimized again using the steepest descent followed by the conjugate gradient method, until the maximum force was smaller than 100 kJ mol^−1^ nm^−1^. The simulation was conducted by using the leap-frog algorithm^50^ with a time-step of 2 fs. The initial atomic velocities were generated according to a Maxwell distribution at 300 K. An equilibration simulation with positional restraint (using a force constant of 1000 kJ mol^−1^ nm^−2^) was carried out for 100 ps, followed by a production run of 20 ns. The simulation was done under the constant NPT condition.

By modifying the weak coupling method^51^, the component of velocity in the essential subspace was coupled to a high temperature of 700 K while the remaining velocity was coupled normally to 300 K. The pressure was coupled to 1 bar with a relaxation time of 0.5 ps and the compressibility of 4.5 × 10^−5^ bar^−1^. All the bonds in the protein were constrained using the P-LINCS algorithm^52^. Twin range cut-off distances for the van der Waals interactions were set to be 0.9 and 1.4 nm, respectively, and the neighbor list was updated every 10 fs. The long-range electrostatic interactions were calculated by the PME algorithm^53^, with an interpolation order of 4 and a tolerance of 10^−5^. The six slowest modes were defined as an essential subspace. Collective modes were updated every 100 time steps according to the new generated protein conformation.

### Fluorescence labeling and spectroscopy

For fluorescence lifetime spectroscopy, single mutants (Y22X, I77X, Y225X, Y102X, F161X or H174X) of HetR were overexpressed in *E. coli* strain BL21 (DE3), as described in the literature^54^. The following steps in protein purification were the same as those for the native protein. The purified proteins in 200 mM NaCl, 20 mM Tris-Cl, pH 7.8 were identified by Coomassie-stained SDS-PAGE. Protein samples (at a concentration of 1.4 μM) were incubated with equimolar amounts of 21-bp duplex DNA at 4 °C for 30 min. The fluorescence lifetime measurements were performed on a DeltaFlex system (Horiba Scientific, Japan). A 374 nm excitation laser with 8 MHz repetition frequency was used. The emission signal was detected at 450 nm (27 ps resolution). The labeled HetR mutants in complex with DNA were analyzed in the presence or absence of 25 μM PatS6. Samples were incubated for 20 min before spectroscopic analysis at 25 °C. Three measurements of each mutant were recorded for signal averaging. The resulting decays were fitted with three exponential decay components:


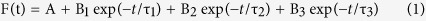


where A is the background offset and B is the pre-exponential function. τ represents lifetime. Amplitude-averaged lifetimes (τ_AV_) for single curve were calculated. All chi-squared values were below 1.20. τ_AV_ for each mutant in each state was represented as mean ± standard error.

## Additional Information

**How to cite this article**: Hu, H. X. *et al.* Structural insights into HetR–PatS interaction involved in cyanobacterial pattern formation. *Sci. Rep.* **5**, 16470; doi: 10.1038/srep16470 (2015).

## Supplementary Material

Supplementary Information

## Figures and Tables

**Figure 1:**
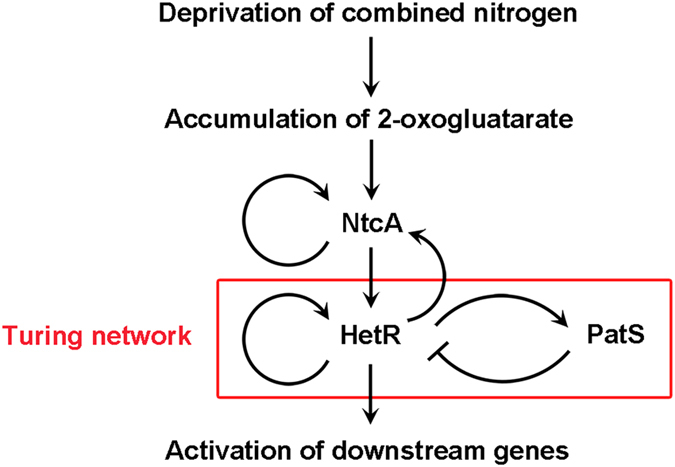
Schematic diagram of the signaling cascade of heterocyst development and pattern formation.

**Table 1:** Data collection and refinement statistics.

	SeMet HetR–DNA	HetR–DNA	PatS6–HetR_Hood_
Data collection			
Space group	*P*4_1_2_1_2	*P*4_1_2_1_2	*C*2
Cell dimensions			
*a*, *b*, *c* (Å)	91.27, 91.27, 243.11	90.56, 90.56, 242.32	218.22, 43.46, 55.11
α, β, γ (°)	90.00	90.00	90.00, 97.54, 90.00
Resolution (Å)	48.62-3.10 (3.27-3.10)	50.00-2.80 (2.90-2.80)	50.00-2.10 (2.15-2.10)
*R*_merge_	13.9 (67.5)	8.2 (53.5)	13.8 (51.7)
*I*/σ*I*	11.5 (3.7)	28.7 (6.8)	7.8 (2.4)
Completeness (%)	99.9 (100)	99.4 (100)	97.5 (95.1)
Redundancy	10.7 (11.2)	14.1 (14.6)	2.9 (2.4)
Refinement			
Resolution (Å)		50.00–2.80	50.00–2.10
No. reflections		25,553	29,699
*R*_work_/*R*_free_		20.3/26.4	21.4/25.6
No. atoms			
Protein		4752	2606
Nucleic acid		861	
Ligand/ion		4	180
Water		31	172
*B*-factors			
Protein		89.86	74.39
Nucleic acid		65.14	
Ligand/ion		103.54	65.07
Water		73.20	44.19
R.m.s. deviations			
Bond lengths (Å)		0.007	0.012
Bond angles (°)		1.256	1.378
PDB entry		4YRV	4YNL

Values in parentheses are for highest-resolution shell.
