# Supplementary material for: Structural insights into HetR−PatS interaction involved in cyanobacterial pattern formation
Source: Sci Rep. 2015 Nov 18;5:16470. doi: 10.1038/srep16470 (PMC4649674; doi:10.1038/srep16470)
Supplement: Supplementary Information [file srep16470-s1.pdf]

Supplementary Information

Structural insights into HetR−PatS interaction involved in cyanobacterial pattern formation

Hai-Xi Hu[1], Yong-Liang Jiang[1], Meng-Xi Zhao, Kun Cai[1], Sanling Liu[1], Bin Wen[1], Pei Lv[1], Yonghui Zhang[1], Junhui Peng[1], Hui Zhong[1], Hong-Mei Yu[1], Yan-Min Ren[1], Zhiyong Zhang[1], Changlin Tian[1], Qingfa Wu[1], Mikael Oliveberg[2], Cheng-Cai Zhang[3], Yuxing Chen[1], and Cong-Zhao Zhou[1]

[1]Hefei National Laboratory for Physical Sciences at the Microscale and School of Life Sciences, University of Science and Technology of China, Hefei Anhui 230027, China. [2]Department of Biochemistry and Biophysics, Arrhenius Laboratories of Natural Sciences, Stockholm University, S-106 91 Stockholm, Sweden. [3]Aix Marseille Université and Laboratoire de Chimie Bactérienne, Unité Mixte de Recherche 7283, Centre National de la Recherche Scientifique, 31 Chemin Joseph Aiguier, 13402 Marseille Cedex 20, France.

Correspondence should be addressed to C.Z.Z. (email: zcz@ustc.edu.cn), Y.C. (email: cyxing@ustc.edu.cn) or C.C.Z. (email: cczhang@imm.cnrs.fr).

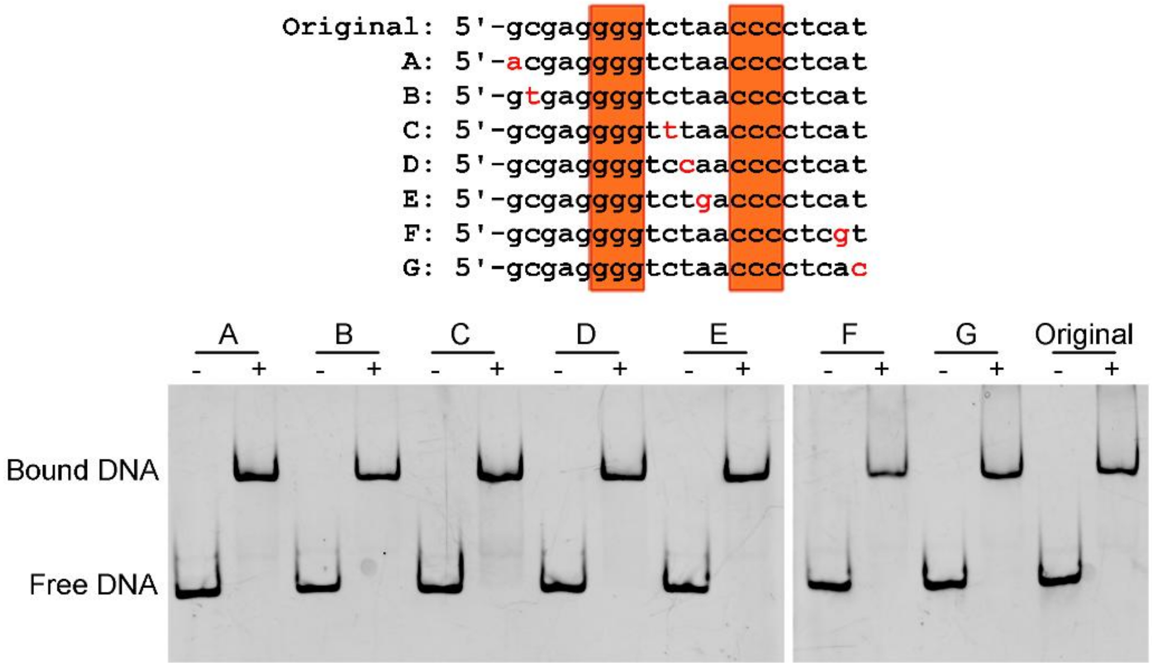

Supplementary Figure 1

Electrophoretic mobility shift assays (EMSA) of HetR with variations of DNA. The DNA samples with and without HetR are shown as "+" and "-", respectively. The corresponding DNA variations are listed above (A to G). Altered bases are shown in red. The 5'-g^{6}gg and 5'-c^{14}cc motifs are highlighted in dark orange.

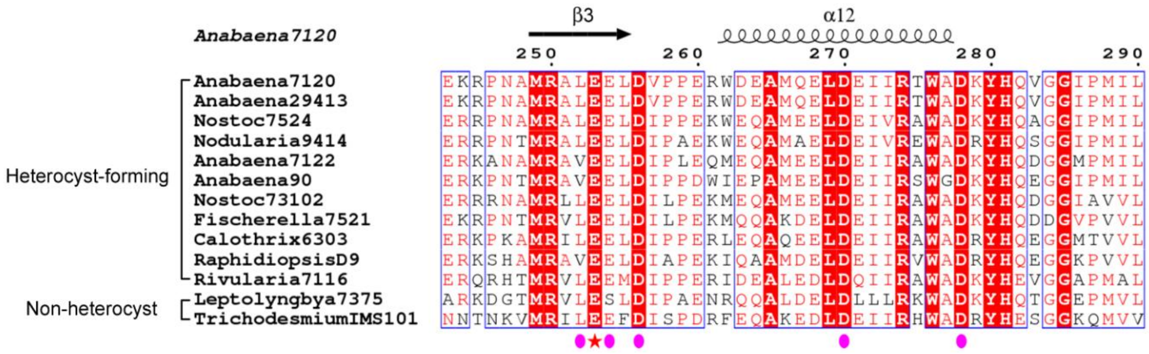

Supplementary Figure 2

Structure-based sequence alignment of the PatS-binding regions of HetR and homologs. The key residue Glu253 for PatS6 binding is indicated by a red star, whereas the other interacting residues are marked with magenta dots. The sequences are from the following cyanobacteria: *Anabaena* 7120, *Anabaena* sp. PCC 7120 (or *Nostoc* sp. PCC 7120); *Anabaena* 29413, *Anabaena variabilis* ATCC 29413; *Nostoc* 7524, *Nostoc* sp. PCC 7524; *Nodularia* 9414, *Nodularia spumigena* CCY9414; *Anabaena* 7122, *Anabaena cylindrica* PCC 7122; *Anabaena* 90, *Anabaena* sp. 90; *Nostoc* 73102, *Nostoc punctiforme* PCC 73102; *Fischerella* 7521, *Fischerella thermalis* PCC 7521; *Calothrix* 6303, *Calothrix* sp. PCC 6303; *Raphidiopsis* D9, *Raphidiopsis brookii* D9; *Rivularia* 7116, *Rivularia* sp. PCC 7116; *Leptolyngbya* 7375, *Leptolyngbya* sp. PCC 7375; *Trichodesmium* IMS101, *Trichodesmium erythraeum* IMS101.

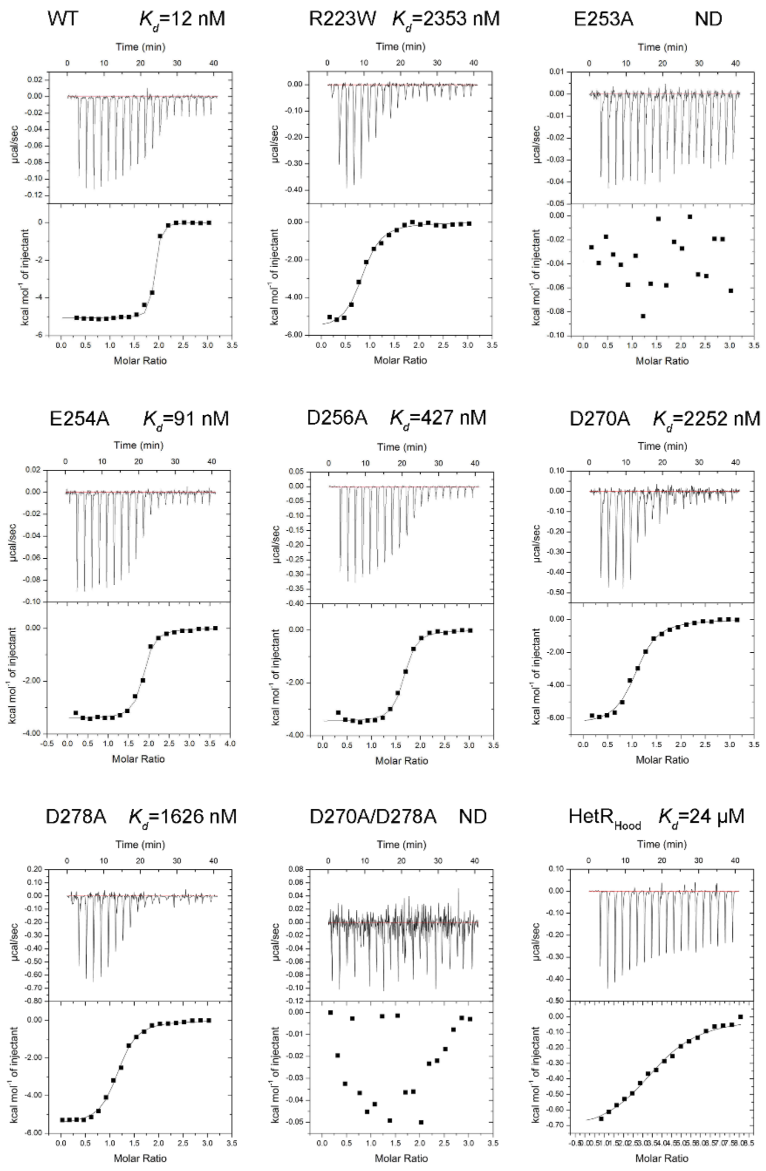

Supplementary Figure 3

The isothermal titration calorimetry (ITC) assays for PatS6 binding to HetR, mutants and HetR$_{\mathbf{Hood}}$. In each figure, the raw data for PatS6 titrated against the protein solution are displayed at the upper panel, and the processed binding isotherms calculated using single binding site model are shown at the lower panel. The related K_d values are calculated and shown above the figures. ND stands for “not detectable”.

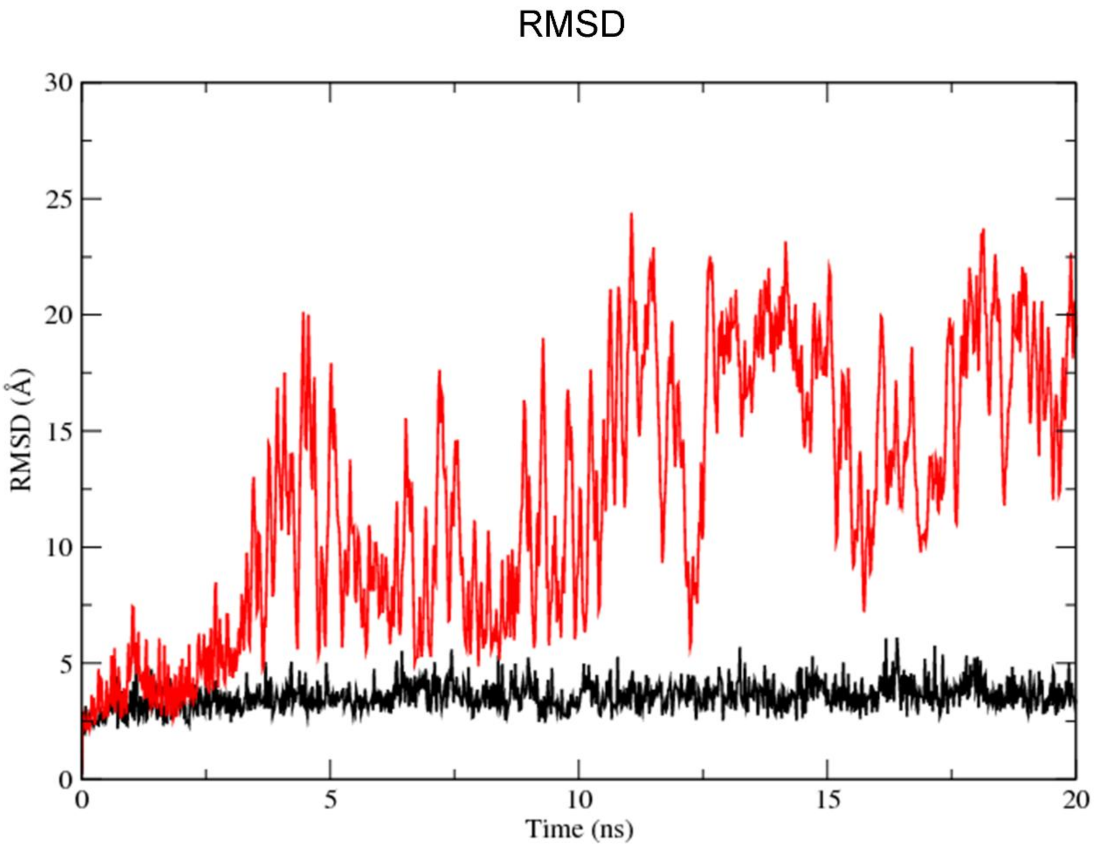

Supplementary Figure 4

Root-mean-square deviation (RMSD) of all 598 Cα atoms of HetR dimer during the 20-ns simulations. The RMSD values from the HetR–DNA and modeled PatS6–HetR–DNA complex are shown as black and red lines, respectively. All structures are superimposed by the DBDs being aligned.

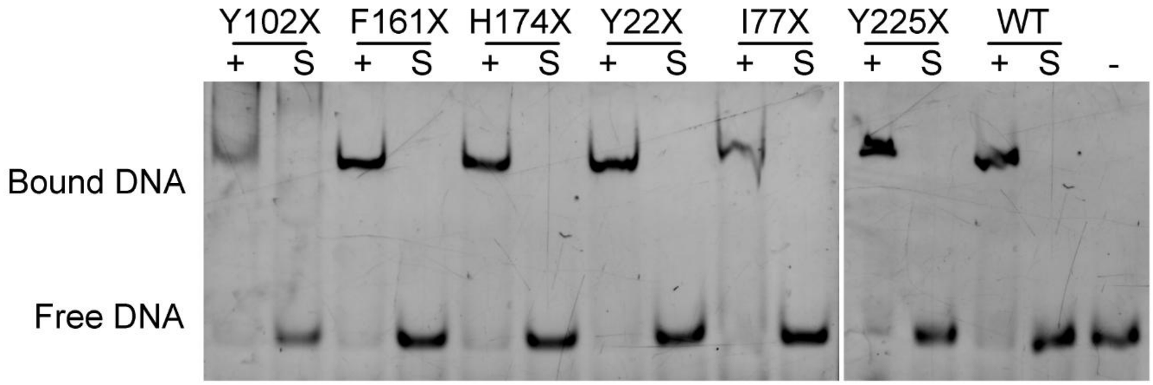

Supplementary Figure 5

EMSA assays of HetR and mutants binding to DNA, in the presence or absence of PatS6. “-” represents DNA probe without protein; “+” indicates DNA incubated with wild-type HetR or mutants; and “S” indicates addition of PatS6 to the HetR−DNA complex.
